# Outcomes following emergent open repair for thoracic aortic dissection are improved at higher volume centers in direct admissions and transfers

**DOI:** 10.1186/s13019-016-0529-5

**Published:** 2016-08-02

**Authors:** Aurelie E. Merlo, Dhaval Chauhan, Chris Pettit, Kimberly N. Hong, Craig R. Saunders, Chunguang Chen, Mark J. Russo

**Affiliations:** 1 Cardiovascular Clinical Research Unit, Barnabas Heart Hospitals, Newark, NJ USA; 2 Case Western Reserve University School of Medicine, Cleveland, OH USA; 3 Department of Surgery, Rutgers - New Jersey Medical School, Newark, NJ USA; 4 Department of Health Evidence and Policy, Mount Sinai School of Medicine, New York, NY USA; 5 Newark Beth Israel Medical Center, Barnabas Heart Hospitals, Newark, NJ USA; 6 Newark Beth Israel Medical Center, Barnabas Health Heart Centers, 201 Lyons Ave, Suite G5, Newark, NJ 07112 USA

## Abstract

**Background:**

The purpose of this study is (1) to define the proportion of patients undergoing emergent open repair of thoracic aortic dissection admitted directly through the emergency room versus those transferred from outside hospitals and (2) to determine if a volume-outcomes relationship exists for those patients across admission types.

**Methods:**

De-identified patient-level data was obtained from the Nationwide Inpatient Sample (2004–2008). Patients undergoing emergent aortic surgery for thoracic aortic dissection (*n* = 1,507) were identified by ICD-9 codes and stratified by annual center volume into low volume (≤5 cases/year) (*n* = 963; 63.9 %), intermediate volume (6–10 cases/year) (*n* = 370; 24.5 %), and high volume (≥11 cases/year) (*n* = 174; 11.6 %) groups. The analysis was further stratified by admission type: direct admission (DA), transfer admission (TA), and other. The primary outcome was in-hospital mortality. Multivariate logistic regression analysis was performed comparing outcomes between high vs low and high vs intermediate volume centers.

**Results:**

Overall in-hospital mortality was 21.8 % (*n* = 328/1,507). Absolute percent mortality at high volume centers was significantly lower (12.6 %) than at medium (20.6 %) and low volume (23.9 %) centers. For DA patients, mortality was 10.6, 21.4, and 24.0 % for high, medium, and low volume centers respectively. For TA patients, mortality was 10.2, 12.7, and 23.5 % for high, medium, and low volume centers, respectively. Multivariate analysis suggested that patients in low volume center were more likely to die compared to high volume center (Odds Ratio 2.06, 95 % CI 1.25 – 3.38, *p* = 0.004). Admission source was not associated with increased mortality.

**Conclusions:**

Direct admissions comprise the largest proportion of dissections regardless of volume strata, and they comprise the largest proportion in the low and intermediate volume cohorts. Admission to low volume center is an independent risk factor for increased mortality. Patients transferred to high volume centers from low volume centers have similar outcome as direct admits in terms of mortality.

## Background

Acute aortic dissections are the most common catastrophe of the aorta [1]. Approximately 2,000 new cases of acute thoracic aortic dissection (ATAD) are reported each year in the United States [2], and they are accompanied by extremely high morbidity and mortality. The risk of mortality is estimated to increase at a rate of 1–2 % per hour after the initial dissection until emergent surgical repair is performed [3]. International Registry of Acute Aortic Dissections (IRAD) data demonstrates overall in-hospital mortality rates of 27.4 %, with a mortality rate of 58 % among patients with acute dissections not receiving surgery, and a mortality rate of 26 % among patients undergoing surgical intervention [4].

Numerous studies have demonstrated a volume-outcomes relationship for complex surgical procedures [5–7]. Luft and colleagues first described this volume-outcomes relationship in cardiac surgical procedures in 1979 with coronary artery bypass grafting. The observation was then subsequently expanded to other cardiothoracic procedures [8–10], including emergent repair for acute aortic dissection [11]. One proposed explanation for better outcomes at high volume centers is survival bias, especially in the setting of emergent procedures. Because high volume centers accept a greater proportion of transfer patients, who are able to survive the added duration of a transfer, those centers may be treating patients who are already pre-selected to have improved outcomes.

To control for potential survival bias at high volume centers, this analysis stratified patients by center volume and admission type. If high volume centers only have improved mortality with Transfer Admits (TA), but not Direct admits (DA), than the reduction in mortality may be due to survival bias. The purpose of this study is to determine if the volume-outcomes relationship persists for patients undergoing emergent open repair for thoracic aortic dissection, at high volume centers, admitted directly through the emergency room and those transferred from other hospitals. We hypothesized that higher volume centers would have lower mortality for both direct admits and transfer admits.

## Methods

### Study population

The Nationwide Inpatient Sample (NIS), which is sponsored by the Agency for Healthcare Research and Quality Healthcare Cost and Utilization Project, was used to identify patient discharges related to emergent aortic dissection repair that occurred between January 1, 2004 and December 31, 2008. The total sample size included 1,507 patients. Because the NIS provides only de-identified patient claims data, this analysis qualified for Institutional Review Board exemption.

The NIS is a 20 % sampling of abstracted discharge data from a national survey of all non-federal acute-care hospitals in the United States, and contains discharge records from over 1,000 hospitals [12]. The database contains up to 15 procedure codes per patient using the International Classification of Diseases, Ninth Revision, Clinical Modification (ICD-9-CM) procedure code index. Acute aortic dissection surgery was abstracted using diagnostic (441.01: Dissection of aorta, thoracic; 441.03: Dissection of aorta, thoracoabdominal) and procedural (38.35: Resection of vessel with anastomosis, other thoracic vessels; 38.45: Resection of vessel with replacement, thoracic vessels) ICD-9-CM codes, in either the first, second, or third code positions. Only patients with admissions coded as emergent, undergoing open repairs, and ≥ 18 years-old were included in the analysis. Of note, while the diagnostic and procedure codes as well as the emergent status strongly select for Type A acute aortic dissections, aortic aneurysms with rupture and complicated Type B aortic dissections are not excluded from the study population. Further, the NIS does not provide data on repeat procedures or mortality beyond the index hospital admission.

### Volume categories and co-morbidity adjustment

**Fig. 1:**
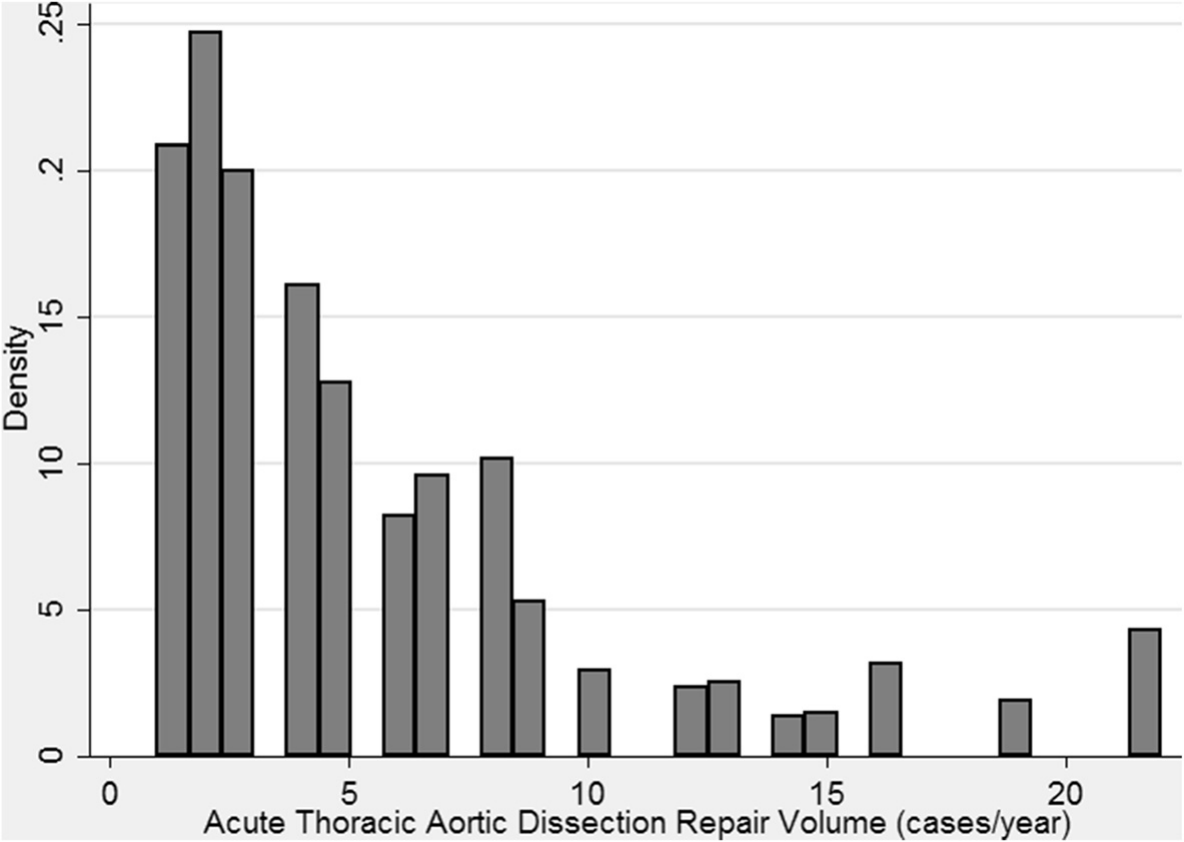
Histogram of case volume distribution across all centers

The average annual distribution of acute aortic dissection repairs across all NIS centers over the five year study period demonstrated three general clusters [Fig. 1]. Based on this distribution, and in keeping with other studies [11], the study population was stratified by annual center volume into low volume (≤5 cases/year) (*n* = 963; 63.9 %), intermediate volume (6–10 cases/year) (*n* = 370; 24.5 %), and high volume (≥11 cases/year) (*n* = 174; 11.6 %) groups. The analysis was further stratified by admission type: direct admission (DA), transfer admission (TA), and other. The other category included patients who were referred from outpatient clinics, health maintenance organization patients and patients for whom admission source data could not be obtained. The primary outcome was mortality.

To account for potential differences in comorbidity burden between center volume groups, the All Patient Refined-Diagnosis Related Group (APR-DRG) Severity of Illness classification was used to compare severity of comorbid illness. The score, first developed by 3 M, allows analysis of outcomes across large cohorts for a given diagnostic group [13]. Severity of illness is defined as the extent of organ system derangement or physiologic decompensation for a patient. The APR-DRG severity of illness subclasses are determined by using discharge billing codes and are based on primary and secondary discharge diagnosis, age, and preexisting medical conditions; codes reflecting in-hospital complications are excluded. The four subclasses of disease severity are: minor (1 point), moderate (2 points), major (3 points), and extreme (4 points). APR-DRG risk of mortality classification was used to adjust outcomes for their risk of mortality. This classification is similar to abovementioned APR-DRG severity of illness classification.

### Statistical analysis

Continuous variables were reported as mean ± standard error and were compared using the Student’s *t* test. Categorical variables were reported as percentages and compared using the chi-squared test. Univariate analysis was performed using student *T*-test comparing two groups. Multivariate logistic regression analysis was performed using variables shown in Table 1, including patient co-morbidities, demographics and APRGRG for mortality risk as well as severity of illness. For all analyses, the conventional *p*-value of 0.05 or less was used to determine the level of statistical significance. All reported *p*-values are two-sided. All statistical analyses were performed using Stata 13 (Stata Corp, College Station, TX).

**Table 1:** Baseline characteristics of study population by center volume

	Low volume			Intermediate volume			High volume	
N	963	63.9		370	24.5		174	11.6
Baseline Characteristic	N/mean	%/stdev	*p*-value*	N/mean	%/stdev	*p*-value*	N/mean	%/stdev
Age (years)	59.1	14.8	0.196	59.6	14.5	0.142	57.6	15.6
Male gender	651	67.6	0.926	253	68.4	0.791	117	67.2
Primary payer: Medicare	356	37	0.037	137	37	0.058	50	28.7
Primary payer: Medicaid	80	8.3	0.003	23	6.2	<0.001	27	15.5
Primary payer: Private insurance	391	40.6	0.185	157	42.4	0.437	80	46
Comorbidities								
APR-DRG severity score	3.38	0.66	0.195	3.46	0.63	0.007	3.31	0.61
Hypertension	585	60.8	0.853	218	59	0.568	107	61.5
Peripheral vascular disorder	183	19	0.868	82	22.2	0.486	34	19.5
Obesity	82	8.5	0.218	25	6.8	0.654	10	5.8
Renal failure	81	8.4	0.046	38	10.3	0.014	7	4.0
Diabetes Mellitus	76	7.9	0.849	25	6.8	0.760	13	7.5
Paralysis	55	5.7	0.123	16	4.3	0.413	5	2.9
Hx alcohol abuse	50	5.2	0.991	19	5.1	0.985	9	5.2
Hx drug abuse	39	4.1	0.708	17	5	0.535	6	3.5

*p values compare group to high volume group

## Results

### Baseline demographics

From January 1, 2004 to December 31, 2008 there were a total of 1,507 patients in the NIS registry who underwent emergent open repair of an acute aortic dissection. When categorized by center volume, 63.9 % of cases (*n* = 963) were performed in low volume centers, 24.5 % (*n* = 370) were performed in intermediate volume centers, and 11.6 % (*n* = 174) were performed in high volume centers. Baseline demographics and co-morbidities by center volume category are shown in Table 1. Significant differences between groups included primary payer distribution and APR-DRG Severity of Illness scores, with scores indicating a greater comorbidity burden in the intermediate volume category and low volume category. However, when APR-DRG scores were compared between DA and transfers among different volume groups, there was no statistical difference, as shown in Table 2. Common co-morbidities included hypertension, obesity, peripheral vascular disorder, and history of substance abuse.

**Table 2:** Average APR-DRG severity of illness and risk of mortality scores by center volume and admission type

	Low volume			Intermediate volume			High volume	
	*N* = 963			*N* = 370			*N* = 174	
Admission type	Mean	St.dev	*p*-value**	Mean	St.dev	*p*-value**	Mean	St.dev
APR-DRG severity of illness score								
Direct Admission	3.39	0.64	0.408	3.44	0.67	0.186	3.32	0.64
Transfer Admission	3.35	0.59	0.322	3.38	0.60	0.244	3.24	0.56
Other	3.37	0.72	0.890	3.57	0.57	0.031	3.36	0.64
Total	3.38	0.66	0.195	3.46	0.63	0.001	3.31	0.61
APR-DRG risk of mortality score								
Direct Admission	3.22	0.70	0.126	3.25	0.72	0.099	3.08	0.85
Transfer Admission	3.19	0.67	0.192	3.14	0.56	0.321	3.02	0.72
Other	3.29	0.73	0.242	3.43	0.64	0.015	3.17	0.65
Total	3.23	0.75	0.015	3.28	0.68	0.004	3.09	0.75

**versus high volume

### Admission types

**Fig. 2:**
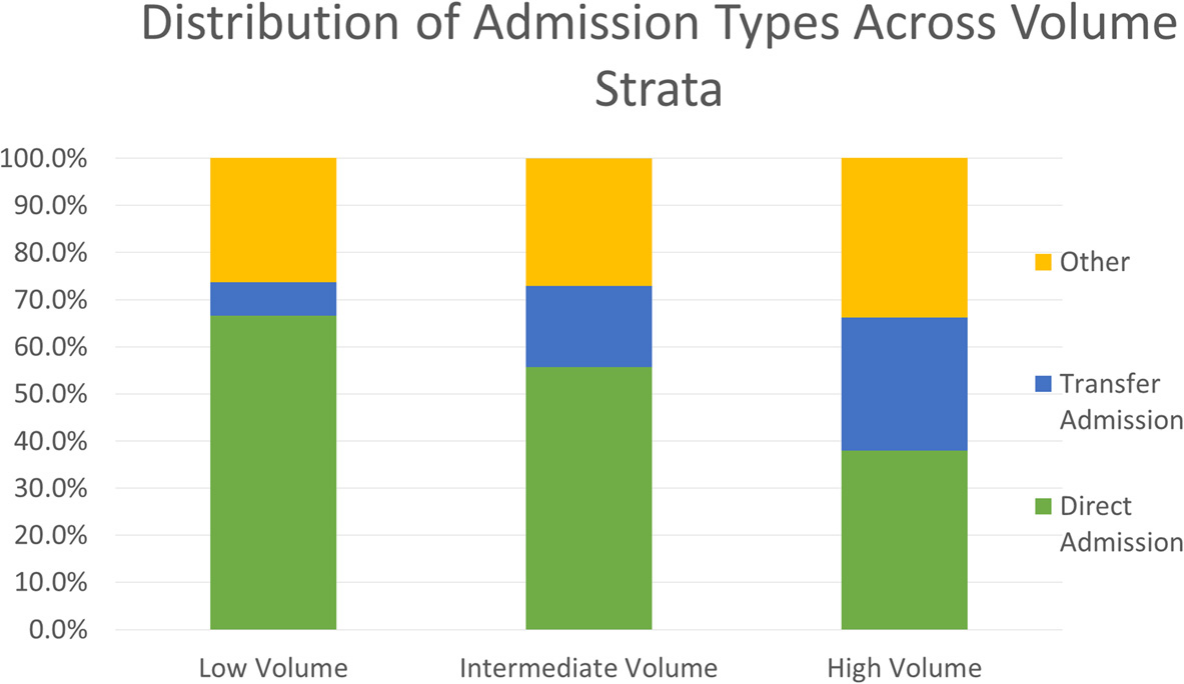
Distribution of admission types by center volume

Average number of admissions in high volume centers was 17.40 ± 9.59 (mean ± SD), intermediate volume center was 9.74 ± 4.71 and in low volume centers was 2.83 + 2.29. Distribution of admission types across the three volume strata are represented in Fig. 2. As expected, high volume centers had a greater proportion of TA (TA) (28.2 %) than intermediate (17.3 %) and low volume (7.1 %) centers. High volume centers, conversely, had a smaller percentage of DA (38.0 %) than intermediate (55.7 %) and low volume (66.6 %) centers.

### Mortality

Mortality data was missing for one patient in the intermediate volume group. Mortality for the entire patient sample was 21.8 % (*n* = 328). Univariate analysis suggested that mortality at high volume centers was significantly lower (22 out of 174 (12.6 %)) than at medium (76 out of 369 (20.6 %), *p* = 0.001) and low volume (230 out of 963 (23.9 %), *p* = 0.025) centers. For DA patients, mortality was 10.6 % (7 out of 66), 21.4 % (44 out of 206), and 24.0 % (154 out of 641) for high, medium, and low volume centers respectively. This demonstrated a statistically significant difference between high and low volume centers (*p* = 0.013), and a difference approaching statistical significance between high and intermediate volume centers (*p* = 0.051). For TA patients, mortality was 10.2 % (5 out of 49), 12.7 % (8 out of 64), and 23.5 % (16 out of 68) for high, medium, and low volume centers, respectively. Differences in mortality between volume strata in transfer patients did not reach statistical significance, due to small sample size. Mortality was lowest at high volume centers for every admission type [Table 3].

**Table 3:** Mortality by center volume and admission type

	Low volume			Intermediate volume			High volume	
	*N* = 963			*N* = 369			*N* = 174	
Admission type	Death/no of patients in group	%	*p*-value**	Death/no of patients in group	%	*p*-value**	Death/no of patients in group	%
Direct Admission	154/641	24.0	0.013	44/206	21.4	0.051	7/66	10.6
Transfer Admission	16/68	23.5	0.064	8/64	12.7	0.683	5/49	10.2
Other	60/254	23.6	0.268	24/100	24.0	0.295	10/59	17.0
Total	230/963	23.9	0.001	76/369*	20.6	0.025	22/174	12.6

*N = 369, due to missing mortality data for 1 patient; *p*-values use high volume group as control group

**versus high volume

Multivariate logistic regression analysis showed that compared to high volume centers, being in low volume centers was an independent risk of mortality with Odds ratio (OR) of 2.06, 95 % Confidence Interval (CI) 1.25 – 3.38, *p* = 0.004. Direct and transfer admissions were not independent risk factors mortality. Other admissions when taken as a variable in multivariate analysis, was collinear with mortality and was omitted from analysis. Other independent risk factors for increased mortality were increased age and increasing APR-DRG mortality risk. Intermediate volume centers were not independent risk factor for increased mortality. See Table 4 for full details.

**Table 4:** Results of multivariate logistic regression analysis comparing outcomes between (1) high vs. low volume centers and (2) high vs. intermediate volume centers

Variable	Odds ratio	Standard error	95 % confidence interval	*p*-value
High volume vs. low volume centers				
Low volume	2.06	0.52	1.25 – 3.38	0.004
Direct admission	1.02	0.17	0.73 – 1.43	0.907
Transfer admission	0.99	0.29	0.56 – 1.75	0.964
Other admission	1.00	0.00	N/A	1.000
Age	1.02	0.01	1.01 – 1.03	0.001
APRDRG risk of mortality	2.20	0.36	1.60 – 3.03	<0.001
High volume vs. intermediate volume centers				
Intermediate volume	1.45	0.41	0.83 – 2.54	0.195
Direct admission	0.90	0.24	0.53 – 1.52	0.692
Transfer admission	0.58	0.22	0.27 – 1.22	0.152
Other admission	1.00	0.00	N/A	1.000
Age	1.03	0.01	1.01 – 1.05	0.005
APRDRG risk of mortality	3.61	1.09	2.00 – 6.51	<0.001

## Discussion

These data from the NIS indicate that the mortality for acute thoracic aortic dissection at low volume centers is almost 2.5 times greater than at high volume centers. This analysis demonstrates that differences in admission type alone cannot explain the differences in outcomes and presents three interesting results: (1) mortality of direct admits and transfer admits was similar within volume strata; (2) direct admits at high volume centers had better outcomes than direct admits at low volume centers; and (3) transfer admits at high volume centers had better outcomes than direct admits at low volume centers.

Many factors, other than admission type, may explain the observed volume-outcomes relationship for emergent repair of acute aortic dissection. For instance, high volume centers have more experienced surgical and non-surgical medical professionals, as well as more streamlined internal processes of care [14–16]. High volume centers may also be more likely to institute multi-disciplinary protocols to better handle suspected cases of aortic catastrophes [17]. Furthermore, differences in volume for ATAD repair, are especially pronounced due to the extremely low case volume across all centers (as opposed to coronary artery bypass grafting, for example). Notably, low volume centers only perform 1 to 5 repairs annually, while high volume centers perform at least twice that amount (>10 cases). Therefore, even slight changes in annual procedural volume have a multiplicative effect on institutional experience.

### Secondary findings

Low volume centers had similar mortality rates for both DA and TA (154 out of 641 patients (24.0 %) and 16 out of 68 patients (23.5 %) for DA and TA). A similar observation was made for high volume centers (7 out of 66 patients (10.6 %) and 5 out of 49 patients (10.2 %) for DA and TA). These findings suggest that center volume may be a more important factor in determining outcome than admission type. DAs, despite being potentially more emergent cases, had the same mortality as TAs at high volume centers. Similarly, TAs at low volume centers did not have improved mortality over DAs, despite potentially having a less emergent status. Determining exactly what characteristics of high volume centers confer this mortality advantage in both DAs and TAs, would be useful for improving ATAD repair outcomes across all volume strata. Nonetheless, a large portion of aortic dissections are still repaired in low volume centers with higher mortality. There needs to be a change in mindset of clinicians to transfer patients with acute aortic dissection to high volume centers where experienced surgeons, state of art operative technology and excellent post-operative patient care is available.

The above findings are further confirmed by another important observation: DAs at high volume centers have improved outcomes compared to DAs at low volume centers. A majority of the comorbidities (hypertension, history of substance abuse, diabetes, and peripheral vascular disease), were evenly distributed across groups with similar APR-DRG scores. Furthermore, comparing DAs at high volume centers and DAs at low volume centers accounts for the survivorship bias associated with transfer admission, as both patient groups are directly admitted to the treating hospital. One possible explanation for the inferior outcomes at low volume centers, is that low volume centers may have a greater diagnostic delay before identifying the ATAD. One study even demonstrates that the delay in diagnosis at a non-tertiary care center is more significant in causing delay for treatment than the delay associated with transferring the patient to a tertiary care center [18]. It is conceivable that the difference we observed in mortality across volume strata may best be explained by a difference in admission to diagnosis time, rather than a difference in diagnosis to open repair time related to patient transfer.

Additionally, TAs at high volume centers were observed to have improved mortality over DAs at low volume centers. While, survival bias is likely a confounding factor, this observation suggests that patients who are able to survive a transfer, may have improved mortality, if transferred to a high volume center, than if they stay at their original hospital.

### Implications

The evidence from this analysis supports efforts for the regionalization of ATAD repairs. Recently, Harris and colleagues conducted a prospective analysis of a standardized, quality-improvement protocol for the regional treatment of acute aortic dissection [19]. The authors demonstrated a reduction in the length of time to diagnosis and surgical repair, increased use of beta-blockers on arrival and discharge, increased use of intraoperative transthoracic echocardiography, and a small decrease in short-term mortality. Despite this study, and the growing body of evidence demonstrating superior outcomes at high volume centers, nearly 65 % of emergent aortic dissection repairs in our analysis were done at centers performing 5 or fewer of such cases each year. As such, the regionalization of care for high-risk aortic surgery, represents an opportunity to improve outcomes for ATAD.

Regionalization has already been proven to be an effective method for improving outcomes, the most prominent example being ST-elevation myocardial infarction (STEMI) networks. Studies have demonstrated improved outcomes after regionalization [20, 21], despite significant barriers to implementation (competition between providers, lack of funding, lack of data collection and unified transfer center protocols, and lack of bed availability) [22, 23]. Today, in the United States, a STEMI center exists for every 585,135 persons [24], and the successes of STEMI networks have generated similar regionalization initiatives for stroke care [25, 26].

However, there is still too few data to expand regionalization practices of more common pathologies, such as STEMIs, to rarer events, such as ATADs. Recent studies analyzing rare and acute events may be beneficial in developing future regionalization networks specific to ATAD. For example, after reports of improved outcomes following subarachnoid hemorrhage repair at high volume centers [27], one group established a critical center volume threshold for subarachnoid hemorrhage repair of 6 cases/year [28]. A similar threshold could be determined in an initial phase of regionalization of ATAD care. Furthermore, these types of analyses could be further extended to other acute events, such as cardiogenic shock, acute respiratory failure, and pulmonary embolus. The regionalization model for each pathology would vary depending on where the pathology fell on the incidence spectrum of rare event (ATAD) to relatively common event (STEMI).

### Limitations

There are several limitations to this analysis. Firstly, the NIS is an administrative database and does not contain detailed patient characteristics or operative variables that may influence outcomes. Specifically, as previously stated, aortic aneurysms with rupture and complicated Type B aortic dissections cannot be excluded from the analysis. Secondly, the categorization of low, intermediate, and high volume centers was based on breakpoints in the distribution of annual center volume, as there are currently no guidelines defining what constitutes a low versus high volume center. Thirdly, APR-DRG severity scores cannot be compared to clinical risk stratification scores such as the STS score, as they are not calculated preoperatively; rather, they are based on ICD-9 billing codes generated after hospital discharge. Nevertheless, APR-DRG has become a preferred method for assessing severity of illness in the analysis of administrative data [29–33]. Finally, although other studies have shown that surgeon-specific volume often drives the volume-outcomes relationship at an institutional level [34], the NIS does not contain data on surgeon-specific volume. This analysis is, therefore, not able to determine whether institutional experience, surgeon experience, or both explain the lower mortality at high volume centers. Our analysis only focused on open aortic aneurysm repairs and therefore its results are not applicable to newly emerged endovascular and hybrid approaches for treatment of aortic dissections.

## Conclusions

Direct admissions comprised the majority of cases of acute aortic dissection in all centers. The volume of direct admits was higher in low and intermediate volume centers. High volume centers have decreased mortality compared to low volume centers for open aortic repair following ATAD, across all admission types. Furthermore, mortality of DAs and TAs was similar for low volume centers and also for high volume centers; DAs at high volume centers had better outcomes than DAs at low volume centers; and TA at high volume centers had better outcomes than DAs at low volume centers. Future studies should seek to prospectively examine processes of care that may explain improved outcomes at high volume centers.

## Abbreviations

APR-DRG: All patient refined-diagnosis related group
ATAD: Acute thoracic aortic dissection
DA: Direct admits (patients admitted directly to the emergency department)
ICD-9-CM: International classification of diseases, ninth revision, clinical modification
IRAD: International registry of acute aortic dissections
NIS: Nationwide inpatient sample
STEMI: ST segment elevation myocardial infarction
TA: Transfer admits (patients admitted from an outside hospital)
